# Therapeutic Overlap Between Bipolar Disorder and Migraine: A Systematic Review of Pharmacological Trials

**DOI:** 10.3390/ph19060848

**Published:** 2026-05-29

**Authors:** Michel Haddad, Luiz Henrique Junqueira Dieckmann, Naielly Rodrigues da Silva, Paula Dieckmann, Thiago Wendt Viola, Jair de Jesus Mari

**Affiliations:** 1 Department of Psychiatry, Brazilian Clinical Research Institute, São Paulo 01404-000, Brazil; michel.psiq@gmail.com (M.H.); luizhjd@gmail.com (L.H.J.D.); naiellyrodriguesdasilva@gmail.com (N.R.d.S.); 2 Department of Psychiatry, Universidade Federal de São Paulo, São Paulo 04021-001, Brazil; paulanatalie@gmail.com; 3 School of Medicine, Pontifical Catholic University of Rio Grande do Sul, Porto Alegre 90619-900, Brazil; thiago.wendt.viola@gmail.com

**Keywords:** bipolar disorder, valproate, valproic acid, migraine

## Abstract

**Background/Objectives**: Migraine is markedly more prevalent among individuals with bipolar disorder (BD) than in the general population. The two disorders share overlapping pathophysiological mechanisms, including neuroinflammation, oxidative stress, and genetic vulnerability. However, the potential bidirectional efficacy of pharmacological agents approved for one condition on the other remains unclear. This study aimed to evaluate cross-disorder pharmacological efficacy between migraine and bipolar disorder. **Methods**: We systematically searched Medline and the Cochrane Central Register of Controlled Trials (PROSPERO registration: CRD420251130780) for randomized controlled trials (RCTs) assessing (1) concurrent treatment effects in comorbid BD–migraine samples, (2) efficacy of migraine treatments in BD, or (3) efficacy of BD treatments in migraine. Searches included guideline-recommended drugs for either disorder, without language or date restrictions. **Results**: A total of 32 RCTs met the inclusion criteria. Fifteen studies evaluated migraine drugs in BD, and sixteen evaluated BD drugs in migraine. No RCTs were identified that simultaneously assessed both conditions within the same sample. Valproate was the only agent demonstrating consistent, replicated efficacy in both conditions, supporting true cross-disorder benefit. Haloperidol and chlorpromazine showed limited evidence for acute anti-migraine efficacy, based solely on placebo-controlled studies, whereas all other guideline-recommended BD drugs lacked evidence of benefit for migraine. Conversely, topiramate, while effective for migraine, was inferior to valproate in BD outcomes, and lamotrigine was effective for BD only when compared with placebo. **Conclusions**: Valproate remains the sole pharmacological agent with robust evidence of bidirectional efficacy in migraine and BD. Most other guideline-recommended medications show disorder-specific effects, highlighting the need for integrative trials addressing pharmacological overlap in comorbid migraine–BD samples.

## 1. Introduction

Migraine is a condition significantly more prevalent in patients with bipolar disorder (BD) compared to the general population, with estimates ranging from 20% to 35% in clinical samples [1,2,3,4]. This comorbidity is clinically meaningful, as patients with both conditions exhibit a higher likelihood of early-onset bipolar symptoms, a greater frequency of depressive episodes, increased rates of anxiety comorbidity, and elevated use of medical services and polypharmacy [5,6]. Both disorders are further conceptualized as episodic, relapsing illnesses characterized by fluctuating symptom trajectories and unpredictable courses, reinforcing the need for long-term therapeutic strategies [7,8,9,10]. These converging observations underscore the need to revisit longstanding assumptions about their separation in clinical practice and to consider whether treatments developed for one condition may exert broader, cross-diagnostic effects.

Migraine and BD share convergent neurobiological pathways that may help explain their frequent co-occurrence and that some pharmacological agents used in one condition may also exert therapeutic effects in the other [11,12]. Both disorders involve alterations in neuronal excitability [13,14], dysregulation of glutamatergic [15,16] and GABAergic [17,18] transmission, and abnormalities in voltage-gated ion channels [19,20].

These overlapping pathophysiological mechanisms [12] provide not only a plausible biological basis for the high comorbidity between migraine and BD but also a potential cross-diagnostic relevance for pharmacological agents such as valproate, topiramate, and lamotrigine, which can modulate some of these shared pathways [21,22,23]. For instance, valproate is characterized by its ability to enhance GABAergic neurotransmission, contributing to a reduction in cortical excitability, an effect that is mediated in part through its actions on sodium and potassium channels [21,24]. Topiramate exhibits a broad pharmacological profile, combining the modulation of sodium and calcium channels with the potentiation of GABAergic activity, as well as the inhibition of excitatory AMPA/kainate receptors [22,25]. In turn, lamotrigine primarily acts by stabilizing neuronal membranes, an effect associated with the inhibition of sodium channels and a consequent attenuation of glutamate release [23,26].

However, despite the high prevalence and clinical relevance of migraine–BD comorbidity, the extent to which pharmacological agents used for one disorder may exert therapeutic effects on the other remains unclear. Existing clinical guidelines for BD and migraine have been developed independently [27,28], with little overlap in evidence synthesis or translational consideration of shared pathophysiological mechanisms [27]. Therefore, the present study systematically reviewed randomized controlled trials (RCTs) to determine whether medications recommended for the treatment of migraine demonstrate efficacy in BD, and conversely, whether guideline-recommended BD medications show efficacy in migraine. We also aimed to identify any available RCT evidence addressing both conditions concurrently within the same study analysis. By mapping cross-disorder evidence from clinical trials, this review aims to provide a comprehensive synthesis of pharmacological overlap and to highlight potential therapeutic targets for patients with co-occurring migraine and BD.

## 2. Methods

The protocol for this systematic review was registered with PROSPERO (International prospective register of systematic reviews), number CRD420251130780. This study was conducted according to the Preferred Reporting Items for Systematic Reviews and Meta-Analyses (PRISMA) 2020 guidelines [29].

### 2.1. Search and Selection

The following electronic bibliographic databases were searched to identify potential studies: MEDLINE (via PubMed) and the Cochrane Central Register of Controlled Trials. No restrictions were applied regarding language or publication year. The search strategy comprised three independent and complementary approaches, designed to capture RCT evidence that may be heterogeneously indexed across bibliographic databases when addressing a cross-disorder research question. Specifically, we sought RCTs that: (1) enrolled patients with comorbid BD and migraine; (2) evaluated guideline-recommended migraine medications in BD populations; and (3) evaluated guideline-recommended BD medications in migraine populations. For approach (1), disease-specific terms were combined with RCT filters—e.g., (“Bipolar Disorder”[MeSH] OR bipolar OR mania) AND (“Migraine Disorders”[MeSH] OR migraine) AND (“Randomized Clinical Trial” OR RCT). For approaches (2) and (3), each search focused on one condition while cross-referencing every guideline-recommended medication for the other. The literature search was conducted in October 2025.

For example, propranolol is a recognized treatment option for migraine. Therefore, we cross-referenced it with terms related to BD ((“Bipolar Disorder”[Mesh] OR Bipolar OR Mania) AND (Propranolol OR Indera) AND (“Randomized Clinical Trial” OR RCT)). Similarly, since lithium is well-recognized and recommended for the treatment of BD, a targeted search strategy was designed to identify RCTs evaluating its efficacy in the treatment of migraine ((“Migraine Disorders”[Mesh] OR Migraine) AND (Lithium) AND (“Randomized Clinical Trial” OR RCT)). Accordingly, a specific search strategy was developed for each drug recommended in guidelines, regardless of whether it was listed as a first-, second-, or third-line treatment option. The reference guideline for BD treatment was the Canadian Network for Mood and Anxiety Treatments (CANMAT) guideline [27], whereas the guideline for migraine treatment was the International Headache Society Global Practice Recommendations for Preventive Pharmacological Treatment of Migraine (IHS) [28]. The full list of individual searches for each drug, along with the corresponding results, is presented in the Supplementary Material.

Eligibility criteria included RCTs enrolling participants diagnosed with BD or migraine according to standardized diagnostic systems, including the Diagnostic and Statistical Manual of Mental Disorders (DSM), the International Classification of Diseases (ICD), or the International Classification of Headache Disorders (ICHD), as applicable. The specific diagnostic framework considered corresponded to the period during which each study was conducted. Given that the included studies covered publications since 1994, accordingly, the DSM-IV (1994) and DSM-5 (2013), as well as ICD-10 (1992) and ICD-11 (2019), were considered eligible, while migraine-specific diagnostic criteria were also based on either ICHD-2 (2004) or ICHD-3 (2018) [30,31,32,33,34,35].

Eligible studies involved pharmacological interventions whose efficacy was clinically evaluated. No restrictions were applied regarding language, publication year, or publication-type indexing. All records retained after de-duplication were evaluated directly at the full-text level by two independent reviewers (NRS and TWV). Both adult and pediatric participants were eligible. Studies addressing all recognized forms of migraine (with aura, without aura, and chronic migraine) were considered. Studies were excluded if they met any of the following criteria: (1) literature reviews; (2) studies focusing on the treatment of psychiatric or neurological conditions other than BD or migraine; (3) studies in which the primary efficacy outcomes were unrelated to BD or migraine symptoms (e.g., evaluating the efficacy of a drug on obsessive–compulsive disorder symptoms in patients with comorbid BD); (4) studies not evaluating a single-drug efficacy (e.g., combined lithium + valproate treatment groups in BD); (5) case reports; or (6) studies focusing solely on cost-effectiveness or pharmacoeconomic analyses.

**Figure 1:**
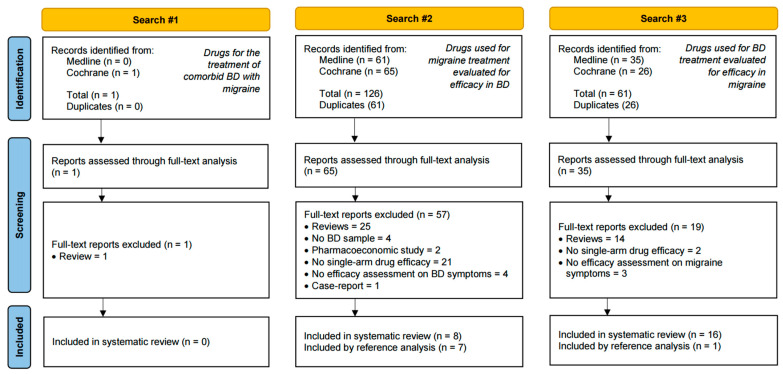
Study selection flowchart diagram. Note: Identification of records across the three search strategies, removal of duplicates, full-text eligibility assessment, reasons for exclusion, and final inclusion of studies in the systematic review. Additional eligible randomized controlled trials identified through reference list screening are also indicated. Abbreviation: BD, bipolar disorder.

Study selection was performed after duplicate removal using Rayyan (Rayyan Systems Inc., Cambridge, MA, USA) [30]. Because each of the three search strategies yielded a small number of records, all retrieved studies were directly evaluated at the full-text level, without a separate title and abstract screening stage. Full-text eligibility assessment was conducted independently by two investigators (NRS and TWV), and reasons for exclusion were recorded for all ineligible reports [36]. Two investigators (NRS and TWV) independently performed these phases. Any disagreements were resolved through discussion with a third reviewer (MH) until consensus was reached. In addition, the reference lists of all included studies were screened to identify additional eligible RCTs, which were also considered for inclusion (Figure 1).

### 2.2. Data Extraction

Following study selection, two independent analysts (TWV and NRS) extracted the data and organized it into a table presenting information from head-to-head comparisons between drugs. Direct comparisons between treatments were analyzed descriptively, based on the primary outcomes of each RCT, without statistical pooling. The table included the first author’s name, year of publication, sample size and treatment arms, treatment clinical phase (for example: acute migraine attacks), dosage, measures of efficacy, outcomes and main findings.

## 3. Results

After removing duplicates, a total of 101 unique records were screened across the three independent search strategies. In the first strategy, all drugs recommended for the treatment of BD or migraine were tested in a search query containing both conditions simultaneously. Only one report was retrieved [37], and no eligible RCTs were identified for inclusion.

The second strategy, which cross-referenced medications used in migraine treatment for potential efficacy in BD, yielded 126 records. After the removal of 61 duplicates, 65 reports were assessed for eligibility, with 8 RCTs meeting the inclusion criteria [38,39,40,41,42,43,44,45]. In addition, screening the reference lists of these studies identified 7 additional RCTs that were also included (*n* = 15) [46,47,48,49,50,51,52].

The third strategy, which examined BD treatments for potential efficacy in migraine, identified 61 records. After removing 26 duplicates, 35 reports were screened, and 16 RCTs were included [53,54,55,56,57,58,59,60,61,62,63,64,65,66,67]. In addition, screening the reference lists of these studies identified one additional RCT that was also included (*n* = 17). In total, 32 RCTs fulfilled all eligibility criteria and were included in the systematic review [38,39,40,41,42,43,44,45,46,47,48,49,50,51,52,53,54,55,56,57,58,59,60,61,62,63,64,65,66,67,68].

### 3.1. Efficacy on Bipolar Disorder of Drugs Used in the Prophylaxis of Migraine

According to the IHS [28], several agents could be prescribed for migraine prophylaxis, including valproate, topiramate, lamotrigine, metoprolol, propranolol, atenolol, bisoprolol, timolol, nadolol, candesartan, lisinopril, flunarizine, amitriptyline, venlafaxine, erenumab, fremanezumab, galcanezumab, eptinezumab, onabotulinumtoxinA, lidocaine, bupivacaine, methylprednisolone, dexamethasone, atogepant, and rimegepant. To investigate the cross-efficacy of these agents in BD, we systematically identified RCTs evaluating their therapeutic effects across different phases of the disorder (mania, depression, and maintenance, clinical phase column in Table 1). The 15 included studies, summarized in Table 1, assessed the efficacy of valproate (*n* = 13) [38,39,40,41,42,43,44,45,46,47,48,50,51], topiramate (*n* = 1) [44], and lamotrigine (*n* = 1) [52].

Valproate was the most extensively evaluated compound, tested across manic, depressive, and maintenance phases in both adult and pediatric samples. Comparators to valproate were: lithium—5 studies [42,47,48,50,51], olanzapine—3 studies [39,40,43], placebo—3 studies [41,45,47], oxcarbazepine—1 study [38], topiramate—1 study [44], and endoxifen—1 study [46]. Across trials, valproate consistently demonstrated significant reductions in manic or depressive symptom severity from baseline to endpoint, as measured primarily by the Young Mania Rating Scale (YMRS) and depression scales such as the Hamilton Depression Rating Scale (HAMD) and the Montgomery–Åsberg Depression Rating Scale (MADRS). In head-to-head comparisons, valproate showed efficacy comparable to lithium in both acute mania and maintenance studies [47,50,51] while also showing superior remission rates, with 72% of patients in the valproate group achieving remission compared to 58% in the lithium group [48]. However, valproate showed inferior results compared to olanzapine in two out of three trials [39,43]. Compared with placebo, valproate demonstrated superior outcomes in reducing manic, depressive, and anxiety symptoms [41,45,49].

Topiramate, evaluated in a single double-blind RCT among adolescents with mania, showed clinical improvement from baseline but was significantly less effective than valproate in reducing YMRS scores [44]. Lamotrigine, assessed in a maintenance-phase study [52], demonstrated similar efficacy to placebo regarding time to additional medication use but significantly reduced relapse rates, with a greater proportion of patients remaining stable until endpoint (41% vs. 26%).

Overall, across all available studies, valproate consistently showed robust efficacy across phases of BD, while lamotrigine exhibited prophylactic benefits during maintenance, and topiramate displayed limited efficacy as an antimanic agent. Detailed study characteristics and main findings are summarized in Table 1.

### 3.2. Efficacy on Migraine of Drugs Used in the Treatment of Bipolar Disorder

According to the CANMAT guideline [27], several drugs are recommended as first-line monotherapy for the treatment of BD, including lithium, quetiapine, valproate, asenapine, aripiprazole, paliperidone, risperidone, and cariprazine. As second-line treatment options, olanzapine, carbamazepine, ziprasidone, and haloperidol are recommended. Finally, as third-line options, chlorpromazine, clozapine, and tamoxifen are recommended. Therefore, we investigated whether RCTs are available that tested these drugs for the treatment of migraine-related disorders. We identified 17 studies, comprising 14 RCTs of valproate [53,54,55,56,57,58,59,60,61,62,63,64,68], 2 RCTs of chlorpromazine [65,66], and 1 RCT of haloperidol, evaluating their efficacy in migraine treatment [67]. The most frequently assessed outcomes were headache frequency, duration, and severity. A summary of the studies selected can be found in Table 2.

Regarding valproate, its efficacy among migraine patients was evaluated both for the acute treatment of migraine (within hours to days) and as a prophylactic option for the prevention of migraine symptoms in studies with longer follow-ups (weeks to months). Comparators included: dexamethasone—3 studies [53,54,55], propranolol—2 studies [56,57], sumatriptan—2 studies [58,59], placebo—2 studies [60,68], levetiracetam—1 study [57], pregabalin—1 study [61], ibuprofen—1 study [62], melatonin—1 study [60], zonisamide—1 study [63], vitamin B2—1 study [69], and the herbal formulation *Sodae*—1 study [64]. In addition, 3 out of the 13 studies assessed the prophylactic efficacy of valproate in pediatric patients with migraine [56,57,61].

Across all studies, valproate demonstrated significant efficacy in reducing migraine-related outcomes compared with baseline (pre-treatment) assessments. In head-to-head comparisons, valproate was significantly more effective than placebo, levetiracetam, propranolol, ibuprofen, sumatriptan, and dexamethasone in some studies. Specifically, the superiority of valproate was evidenced by greater overall efficacy relative to placebo [60,68]; higher complete remission rates compared with levetiracetam and propranolol [57]; greater frequency of pain relief compared with ibuprofen, defined as at least a 50% reduction in pain intensity [62]; faster treatment response compared with sumatriptan [58], with significant improvement in photophobia, phonophobia, nausea, and vomiting, whereas only photophobia and vomiting improved significantly with sumatriptan [59]; and significant improvement of acute headache among patients with aura, whereas dexamethasone showed no such effect [55]. Valproate also showed similar efficacy or inferiority relative to specific comparator medications, and these findings are summarized in Table 2.

Chlorpromazine was evaluated for its efficacy in the acute treatment of migraine, with comparators including: placebo—1 study [65], dexamethasone—1 study [66], ketorolac—1 study [66], and metoclopramide—1 study [66]. Chlorpromazine was significantly superior to placebo across all assessed outcomes; however, its efficacy was comparable to that of the other comparator drugs [66]. Finally, haloperidol was tested for its efficacy in the acute treatment of migraine against placebo (1 study), demonstrating significant superiority [67].

### 3.3. Synthesis of Findings

**Figure 2:**
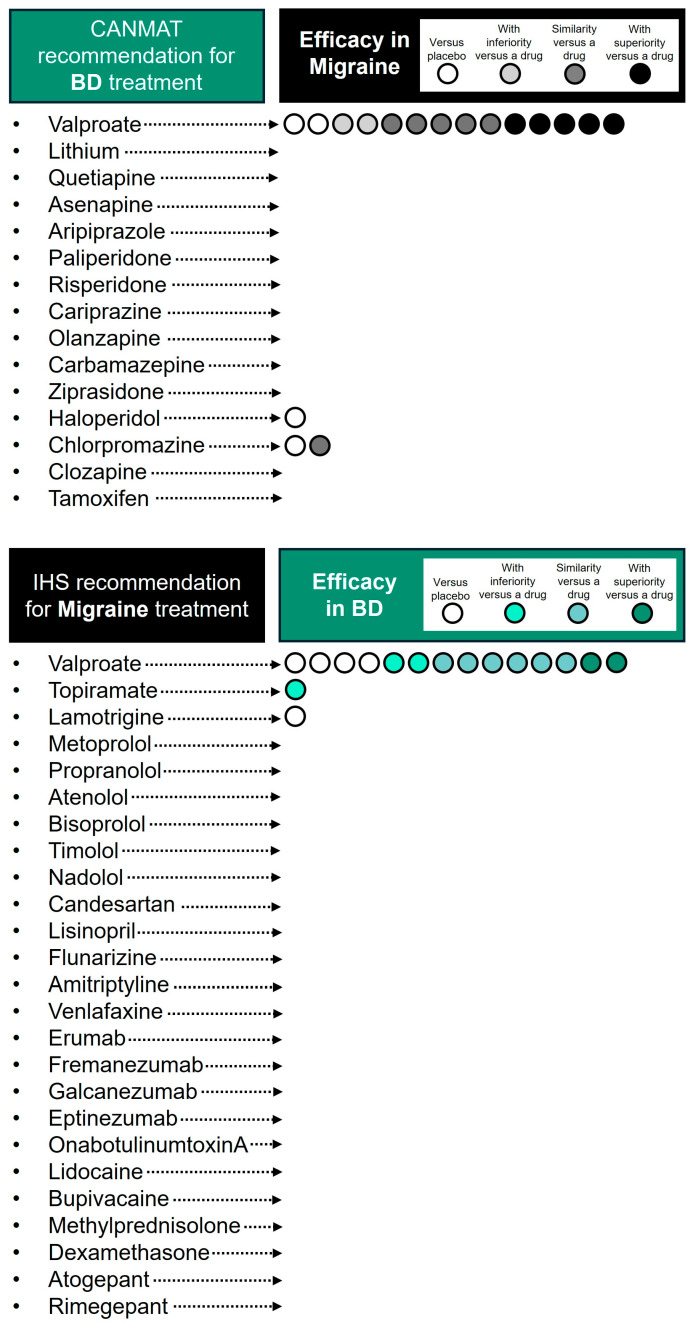
Integrated overview of pharmacological agents recommended by CANMAT for bipolar disorder and by IHS for migraine, displaying the available RCT evidence for each drug’s efficacy in the alternate condition. Note: The number and color of circles represent the number of RCTs reporting each type of evidence (versus placebo, with inferiority, similarity, or superiority versus another drug). Abbreviations: BD: Bipolar Disorder; CANMAT: the Canadian Network for Mood and Anxiety Treatments; HIS: the International Headache Society Global Practice Recommendations for Preventive Pharmacological Treatment of Migraine. The figure was generated manually using Microsoft PowerPoint.

Figure 2 summarizes the cross-efficacy between pharmacological agents recommended by current guidelines for migraine and BD treatment. Among guideline-recommended BD drugs, valproate was the only compound showing consistent efficacy in migraine, with multiple trials demonstrating superiority or equivalence to active comparators and clear benefit over placebo. Haloperidol and chlorpromazine presented limited evidence of acute anti-migraine efficacy, particularly versus placebo. All other BD agents, including lithium, quetiapine, olanzapine, risperidone, aripiprazole, carbamazepine, ziprasidone, clozapine, and tamoxifen, lacked evidence of efficacy on migraine outcomes in RCTs.

Among drugs recommended for migraine prevention, valproate again demonstrated robust efficacy in BD, confirming its bidirectional therapeutic value. Notably, five trials demonstrated the superiority of valproate over placebo or other pharmacological agents in the treatment of BD [41,44,45,48,49]. Topiramate and lamotrigine exhibited asymmetric profiles: topiramate was effective for migraine but not for BD, particularly when compared to valproate, whereas lamotrigine was effective for BD but only when compared to placebo. All remaining guideline-recommended migraine medications, including β-blockers, antihypertensives, antidepressants, CGRP monoclonal antibodies, onabotulinumtoxinA, local anesthetics, corticosteroids, and gepants, lacked evidence of efficacy in BD based on RCTs.

## 4. Discussion

This systematic review used three complementary search strategies to identify pharmacological overlap between migraine and BD. No RCTs evaluating agents simultaneously in both conditions were identified.

When migraine treatments were examined for efficacy in BD, 15 RCTs met the inclusion criteria, largely driven by valproate, which showed consistent benefits across manic, depressive, and maintenance phases. Conversely, 17 RCTs assessed BD treatments for migraine outcomes, again predominantly involving valproate, which was the only agent demonstrating reproducible efficacy in both acute and preventive migraine settings. Overall, across 32 RCTs, the findings indicate a limited therapeutic overlap between migraine and BD, with valproate emerging as the sole medication with consistent and bidirectional efficacy across both disorders.

Valproate, commonly prescribed as an anticonvulsant, is also widely used as a mood stabilizer [70]. In bipolar disorder, a study evaluating the prescription patterns of pharmacotherapy evidenced that valproate was the most prescribed mood stabilizer [71], reflecting its superiority to placebo and its equivalence to lithium across different phases of BD [72,73]. Also, an overview of 26 systematic reviews and meta-analyses further demonstrated that valproate is more effective than placebo for the treatment of acute mania, bipolar depression, and the maintenance phase of treatment [72]. Among guideline-recommended migraine agents evaluated in BD, valproate consistently demonstrated benefit across manic, depressive, and maintenance phases, with effects comparable to those of lithium and superior to placebo [72,73]. Conversely, topiramate and lamotrigine, two migraine prophylactic agents with mechanistic overlap, did not demonstrate meaningful cross-disorder efficacy: topiramate was consistently inferior to valproate or placebo in BD trials [44,74,75,76,77], and lamotrigine, while effective in bipolar depression, lacked trial evidence in migraine populations [52,78,79].

Similarly, when BD treatments were evaluated for migraine, valproate again showed consistent benefit. Across acute and prophylactic trials, valproate reduced headache frequency and severity and, in several studies, performed as well as or better than active comparators [80,81]. These findings are concordant with the most recent clinical guidelines from the American College of Physicians, which list valproate as a first-line preventive therapy for migraine in adults [82]. A systematic review and meta-analysis comparing preventive pharmacological approaches for migraine reported moderate-quality evidence indicating that valproate increases the proportion of patients achieving a 50% or greater reduction in monthly migraine days [80]. Even at low serum levels, a significant reduction in migraine attacks has been reported [81]. Moreover, compared to other treatment options, valproate has shown similar efficacy for the prevention of migraine in pediatric patients [83].

Other guideline-recommended BD agents showed limited evidence of benefit on migraine. Haloperidol and chlorpromazine demonstrated short-term anti-migraine efficacy in acute emergency-department settings, likely reflecting the analgesic and antiemetic effects of dopamine blockade, but these agents lack support for long-term or preventive use [84,85,86]. No atypical antipsychotic showed cross-efficacy in migraine trials.

Mechanistically, valproate is a multimodal agent whose principal actions include the enhancement of GABAergic neurotransmission and the modulation of calcium, sodium, and potassium channels. Converging evidence indicates that disturbances in GABAergic tone may represent a shared biological substrate between migraine and mood disorders. In migraine, reduced GABA concentrations have been reported in the anterior cingulate cortex and prefrontal cortex of patients without aura [17]. Also, it has been demonstrated that depressed patients have a highly significant reduction, approximately 52%, in occipital cortex GABA levels compared with healthy controls [87]. By enhancing inhibitory GABAergic signaling, valproate may counteract these deficits, thereby mitigating mood instability in BD and reducing susceptibility to migraine attacks.

The ion channels targeted by valproate are also implicated in the physiopathology of migraine and BD [88]. Genetic variations of sodium channels contribute to familial hemiplegic migraine [89]. Voltage-gated calcium channels are involved in migraine initiation [90] and calcium channels blockers might be effective in migraine management [91]. Valproate’s inhibitory effects on these channels are thought to dampen excessive neuronal firing and cortical spreading depolarization, mechanisms closely linked to migraine generation. In relation to BD, genome-wide association studies have consistently highlighted calcium-channel involvement in bipolar disorder, with *CACNA1C*, particularly the intronic SNP rs1006737, emerging as the most robust susceptibility locus across multiple large consortia [92]. Thus, the ability of valproate to act across multiple ion-channel systems may explain why it shows therapeutic effects across manic, depressive, and maintenance phases, as well as across acute and preventive migraine settings.

Also, as in migraine, some evidence suggests that calcium-channel blockers may also hold therapeutic potential in BD [93]. Additional risk variants have been identified in genes such as *ANK3* (rs10994336) and *CACNG4/CACNG5* (rs17645023), which encode proteins critical for sodium-channel assembly and function, further reinforcing ion-channel dysregulation as a core biological feature of the disorder [92]. The concentration of findings indicating beneficial cross-effect around valproate may reflect its broad pharmacologic profile [21,24]. Unlike more selective agents, valproate simultaneously targets several convergent neurobiological pathways, which may underlie its unique and reproducible bidirectional efficacy. Although the exact neuropathological mechanisms differ across BD and migraine, valproate modulates multiple biological processes relevant to both [17,70,88]. These multimodal properties may help explain its unique bidirectional therapeutic signal. Still, mechanistic hypotheses should be interpreted cautiously, as existing RCTs were not designed to evaluate mechanistic pathways directly.

In addition to the pathways that overlap with valproate’s pharmacological profile, other shared neurobiological pathways, such as calcitonin gene-related peptide (CGRP) dysregulation, also link migraine and mood disorders [94]. Treatments targeting CGRP, such as monoclonal antibodies, are used for migraine prevention and have shown promise in potentially improving comorbid depressive symptoms [94,95,96]. However, anti-CGRP monoclonal antibodies are not yet approved for the treatment of bipolar disorder.

From a practical clinical perspective, the consistent bidirectional efficacy observed for valproate suggests that it may be a particularly suitable choice for patients presenting with both bipolar symptoms and recurrent migraine, especially when treatment simplification or cross-condition coverage is desirable. Nonetheless, valproate should be avoided in patients for whom reproductive safety is a major concern and in those with clear contraindications such as significant hepatic impairment [97]. Its use, however, still requires routine attention to tolerability, as common adverse effects such as weight gain, and gastrointestinal discomfort may influence adherence [97,98].

### Limitations

Important limitations should be noted. Evidence outside of valproate was sparse, with most drug classes represented by one or two small trials. This fragmentation limits the ability to discern whether these agents are truly ineffective across diagnoses or whether the apparent lack of benefit reflects insufficient evidence. Additionally, none of the available RCTs specifically enrolled patients with comorbid BD and migraine, a population known to present distinct clinical challenges and potentially different treatment responses [3,6,99,100]. The review relied on two databases (PubMed and the Cochrane Central Register of Controlled Trials), which, although widely used and methodologically robust, may have excluded relevant studies indexed elsewhere. Moreover, the substantial heterogeneity across study designs, outcome measures, and diagnostic criteria precluded the possibility of conducting a quantitative synthesis or pooling the available data, thereby limiting the capacity to generate more robust comparative estimates. Finally, the selection of international guidelines to define first-line and recommended treatments, although grounded in their broad global acceptance, introduces an element of arbitrariness, as guideline recommendations may vary across regions and professional bodies. In addition, the search was restricted to RCTs only, which may have excluded relevant evidence from open-label studies and real-world clinical data. Finally, eligible studies were heterogeneous with respect to participant demographics, and the review did not systematically account for potential variations in treatment effects related to age group, sex, race/ethnicity, or country of origin, limiting the generalizability of the findings to specific patient populations.

Despite these limitations, the findings suggest that valproate holds a unique position among available treatments, showing consistent efficacy independently in both bipolar disorder and migraine and may represent a rational first-line choice when both conditions are present. However, the absence of well-powered, comorbidity-focused RCTs remains a critical gap. Future trials designed specifically for this population are needed to determine whether valproate is truly unique in its cross-diagnostic efficacy or whether additional therapeutic options may emerge with more systematic investigation.

## Figures and Tables

**Table 1:** Evidence for the efficacy of migraine treatments (valproate, topiramate, and lamotrigine) in bipolar disorder.

Reference	Sample Size	Dose of Target Drug	Clinical Phase	Double Blinding	Measure of Efficacy	Main Findings
[38]	Oxcarbazepine (*n* = 13), Valproate (*n* = 13)	Valproate 20 mg/kg/day oral	Mania in adults	Yes	Mania symptom severity (YMRS), clinical global impression (CGI-S)	Both outcomes significantly decreased from baseline to endpoint in both treatments, with no group differences.
[39]	Olanzapine (*n* = 514), Valproate (*n* = 107)	Valproate 400 mg/day oral	Mania in adults	Yes	Mania symptom severity (YMRS), depressive symptom severity (HAMD)	Both groups improved from baseline to endpoint in mania and depressive symptoms, with significantly greater improvements in the Olanzapine compared with the Valproate group.
[42]	Lithium (*n* = 65), Valproate (*n* = 69)	Valproate 15 to 20 mg/kg/day oral	Maintenance in adults	No	Number of months without mania or depression episodes *	The mean number of months without DSM-IV mania and depression was 5.3 for Valproate and 5.4 for Lithium, with no group differences.
[41]	Placebo (*n* = 12), Valproate (*n* = 13)	Valproate 500 mg/day oral	Depression in adults	Yes	Depression symptom severity (HAMD), anxiety symptom severity (HSRA), clinical global Impression (CGI-S)	Valproate was significantly more effective than Placebo in reducing symptoms of depression and anxiety. Patients treated with Valproate had a trend in improvement on the clinical impression compared to those treated with Placebo.
[40]	Olanzapine (*n* = 57), Valproate (*n* = 63)	Valproate 15 to 20 mg/kg/day oral	Mania in adults	Yes	Mania symptom severity (YMRS)	Although both drugs were effective, no significant differences between the treatment groups for baseline-to-endpoint mania symptom severity were observed.
[43]	Olanzapine (*n* = 125), Valproate (*n* = 123)	Valproate 500–2500 mg/day oral	Mania in adults	Yes	Mania symptom severity (YMRS), response, time-to-response, remission, time-to-remission	Although both drugs were effective, baseline-to-endpoint improvements in all mania-related outcomes were significantly greater in the Olanzapine group compared with the Valproate group, except for treatment response.
[44]	Topiramate (*n* = 125), Valproate (*n* = 123)	Valproate 500–2500 mg/day oral	Mania in adolescents	Yes	Mania symptom severity (YMRS)	Baseline-to-endpoint improvements in mania symptoms were significant in both treatments, but were significantly greater in the Valproate group compared with the Topiramate group.
[45]	Placebo (*n* = 9), Valproate (*n* = 9)	Valproate serum concentration 70–90 ng/dL oral	Depression in adults	Yes	Depressive symptom severity (MADRS)	The Valproate treatment group showed significantly greater reduction in depression compared to Placebo.
[46]	Endoxifen (*n* = 28), Valproate (*n* = 14)	Valproate 1000 mg/day oral	Mania in adults	Yes	Mania symptom (YMRS) treatment response	Although both drugs were effective, similar response rates were observed for Endoxifen (64%) and Valproate (72%).
[47]	Lithium (*n* = 36), Valproate (*n* = 69), Placebo (*n* = 74)	Valproate 1000 mg/day oral	Mania in adults	Yes	Mania symptoms (SADS-C) treatment response	Treatment response was greater in Valproate and Lithium compared to Placebo.
[48]	Valproate (*n* = 122), Lithium (*n* = 135)	Valproate 20 mg/kg/day oral	Mania in adults	Yes	Mania symptom severity, response and remission (YMRS)	Similar improvements in mania symptom and response were observed between treatments. However, a significantly greater proportion of patients in the Valproate group (72%) achieved remission compared with those in the Lithium group (58%).
[49]	Placebo (*n* = 28), Valproate (*n* = 26)	No information	Depression in adults	Yes	Depressive symptom severity, response and remission (MADRS)	The Valproate treatment group showed a significantly greater reduction in depressive symptoms and a higher response rate. However, only a statistical trend favoring Valproate was observed for remission (Valproate: 23%; Placebo: 10%).
[50]	Lithium (*n* = 32), Valproate (*n* = 28)	Valproate 1500 mg/day oral	Maintenance in adults	No	Mania or depressive episode relapse, time to relapse	Similar rates of relapse into any mood episode for those given Lithium (56%) versus Divalproex (50%) were observed. There were no significant differences in time to relapse.
[51]	Lithium (*n* = 30), Valproate (*n* = 30)	Valproate serum concentration > 50 ng/dL	Maintenance in children	No	Mania or depressive episode time to relapse	Similar times to any mood episode relapse were observed for Valproate (112 days) and Lithium (114 days).
[52]	Lamotrigine (*n* = 92), Placebo (*n* = 88)	Lamotrigine 100–500 mg/day	Maintenance in adults	Yes	Time to additional medication for emerging symptoms, relapse	No difference between treatment groups in time to additional pharmacotherapy. However, a significant difference was observed for relapse rates, with 41% of patients in the Lamotrigine group remaining stable until the endpoint, compared with 26% in the Placebo group.

Note: Response was defined as at least 50% reduction in symptom severity during follow-up. The study included additional quality-of-life-related outcomes that were not incorporated into our report (*). The ‘Clinical phase’ column indicates the phase of bipolar disorder targeted by each trial, mania (acute treatment of a manic episode), depression (acute treatment of a bipolar depressive episode), or maintenance (long-term prophylaxis aimed at preventing recurrence of mood episodes), together with the age group enrolled (adults, adolescents, or children). Abbreviations: YMRS: Young Mania Rating Scale; HAMD: Hamilton Depression Rating Scale; MADRS: Montgomery–Åsberg Depression Rating Scale; CGI-S: Clinical Global Impression–Severity; SADS-C: The Schedule for Affective Disorders and Schizophrenia–Mania Symptoms.

**Table 2:** Evidence for the efficacy of bipolar disorder treatments (valproate, haloperidol, and chlorpromazine) in migraine.

Reference	Sample Size	Dose of Target Drug	Clinical Phase	Double Blinding	Measure of Efficacy	Main Findings
[57]	Propranolol (*n* = 13), Valproate (*n* = 13), Levetiracetam (*n* = 13)	Valproate 15 mg/kg/day oral	Pediatric migraine prophylaxis	No	Headache symptoms, frequency, remission, and disability (PedMIDAS)	All three drugs significantly reduced the outcomes assessed, with no significant differences between groups. However, complete remission occurred significantly more in the Valproate group (92%) compared to Propranolol (69%) and Levetiracetam groups (30%).
[61]	Pregabalin (*n* = 32), Valproate (*n* = 32)	No information	Pediatric migraine prophylaxis	No	Headache frequency, intensity, duration, remission	Both drugs were equally effective in reducing intensity, duration and frequency. However, complete remission occurred in 42% of the Pregabalin group and 20% in the Valproate group.
[62]	Ibuprofen (*n* = 50), Valproate (*n* = 49)	Valproate 800 mg IV-infusion	Acute migraine attacks in adults	Yes	Pain intensity (NRS) and relief *	Reduction in pain intensity was significantly greater with Valproate. Pain relief was achieved in 100% of Valproate patients versus 60% of Ibuprofen patients.
[54]	Dexamethasone (*n* = 40), Valproate (*n* = 40)	Valproate 400 mg IV-infusion	Acute migraine attacks in adults	Yes	Pain intensity (VAS); success defined as VAS ≤ 3	Both drugs significantly reduced pain, with no intergroup differences.
[60]	Placebo (*n* = 35), Melatonin (*n* = 35), Valproate (*n* = 35)	Valproate 200 mg/day oral	Adult migraine prophylaxis	Yes	Attack frequency, duration, severity, response *, and disability (MIDAS)	Both active drugs significantly reduced all efficacy outcomes compared with Placebo, with no differences between Melatonin and Valproate. Therapeutic response in 34%, 37%, and 20% of patients in the Melatonin, Valproate, and Placebo groups, respectively.
[63]	Zonisamide (*n* = 48), Valproate (*n* = 48)	Valproate 200–600 mg/day oral	Adult migraine management	Yes	Attack severity, duration, frequency	Both drugs were equally effective in reducing migraine attacks.
[58]	Sumatriptan (*n* = 18), Valproate (*n* = 19)	Valproate 15 mg/kg subcutaneous	Acute migraine attacks in adults	No	Pain intensity (VAS), time for onset of action, duration, and recurrence	Although both drugs were effective, the response to treatment in the Valproate group was faster and more pronounced within 30 min and 1 h compared to the Sumatriptan group.
[69]	Vitamin B2 (*n* = 45), Valproate (*n* = 45)	Valproate 500 mg/day oral	Adult migraine prophylaxis	Yes	Headache frequency, duration and severity	Both treatments were similarly effective, with no significant differences between groups.
[55]	Dexamethasone (*n* = 43), Valproate (*n* = 43)	Valproate 400 mg IV-infusion	Acute migraine attacks in adults	Yes	Pain intensity (VAS) in the presence of nausea and photophobia	Both treatments were effective in improvement of acute headache in patients without aura. However, Valproate significantly improved the acute headache in patients with aura but Dexamethasone did not.
[59]	Sumatriptan (*n* = 45), Valproate (*n* = 45)	Valproate 400 mg IV-infusion	Acute migraine attacks in adults	Yes	Pain intensity (VAS)	Valproate and Sumatriptan showed similar efficacy. Photophobia, phonophobia, nausea, and vomiting improved significantly with Valproate, while only photophobia and vomiting improved significantly with Sumatriptan.
[68]	Placebo (*n* = 44), Valproate (*n* = 44)	Valproate 800 mg/day oral	Medication-overuse headache	Yes	Headache frequency, duration and severity, response *, need for rescue medication	The response rate was significantly greater in the sodium valproate group (45.0%) compared with the placebo group (23.8%). Participants receiving valproate also experienced significantly fewer migraine days and reduced use of rescue medications than those receiving placebo.
[53]	Dexamethasone (*n* = 12), Valproate (*n* = 19)	Valproate 900 mg IV-infusion	Acute migraine attacks in adults	Yes	Headache pain severity, time to onset of action and to maximum relief, relapse	Both treatments were effective with no significant differences between them.
[56]	Propranolol (*n* = 58), Valproate (*n* = 57)	Valproate 10 mg/kg/day oral	Pediatric migraine prophylaxis	No	Headache frequency, duration, severity, response *	The two drugs showed similar efficacy in all tested outcomes.
[64]	Sodae Herbal (*n* = 36), Valproate (*n* = 40)	No information	Adult migraine prophylaxis	Yes	Headache frequency, duration, severity, disability (MIDAS)	Both drugs showed efficacy in reducing the frequency, severity and duration of headache. The proportion of patients with severe disability decreased significantly more in the Sodae group compared with the Valproate group.
[67]	Haloperidol (*n* = 58), Placebo (*n* = 60)	Haloperidol 2.5 mg IV-infusion	Acute migraine attacks in adults	Yes	Pain intensity (VAS), and response *	There was a significant greater reduction in pain in the Haloperidol group compared with Placebo group. A response was observed in 34% of patients in the Haloperidol group, compared with 11% in the placebo group.
[66]	Dexamethasone (*n* = 32), Ketorolac (*n* = 32), Metoclopramide (*n* = 32), Chlorpromazine (*n* = 32)	Chlorpromazine 25 mg IV-infusion	Acute migraine attacks in adults	Yes	Pain intensity (VAS), and response *	The effect of all mentioned drugs on acute migraine headache pain severity was significant post-treatment compared to baseline. No significant difference was detected between groups regarding response rates.
[65]	Chlorpromazine (*n* = 32), Placebo (*n* = 60)	Chlorpromazine 0.1 mg/kg IV-infusion	Acute migraine attacks in adults	Yes	Pain intensity, nausea, photophobia, phonophobia, need for rescue medication	Chlorpromazine was significantly superior in all outcomes compared with Placebo. Those receiving Chlorpromazine had less nausea and dyspepsia, but more drowsiness and postural hypotension than those receiving placebo.

Note: *—Pain relief or response was determined based on a decrease of ≥50% in the pain score compared to the baseline. Abbreviations: NRS: Numerical Rating Scale; VAS: Visual Analog Scale; MIDAS: Migraine Disability Assessment; IV: Intravenous.

## Data Availability

The original contributions presented in this study are included in the article/Supplementary Material. Further inquiries can be directed to the corresponding author.

## Supplementary Materials

The following supporting information can be downloaded at: https://www.mdpi.com/article/10.3390/ph19060848/s1. Supplemental Material—Full search strategy.
