# Supplementary material for: Therapeutic Overlap Between Bipolar Disorder and Migraine: A Systematic Review of Pharmacological Trials
Source: Pharmaceuticals (Basel). 2026 May 29;19(6):848. doi: 10.3390/ph19060848 (PMC13305864; doi:10.3390/ph19060848)
Supplement: Supplementary file 1 [file pharmaceuticals-19-00848-s001.zip › pharmaceuticals-4264939-supplementary.pdf]

SUPPLEMENTAL MATERIAL – FULL SEARCH STRATEGY

PUBMED SEARCHES

Strategy #1

("Bipolar Disorder"[Mesh] OR Bipolar OR Mania) AND ("Migraine Disorders"[Mesh] OR Migraine) AND ("Randomized Clinical Trial" OR RCT) = 0

Strategy #2

"Bipolar Disorder"[Mesh] OR Bipolar OR Mania

("Bipolar Disorder"[Mesh] OR Bipolar OR Mania) AND (Valproic acid OR Valproate OR Divalproex) AND ("Randomized Clinical Trial" OR RCT) = **37**

("Bipolar Disorder"[Mesh] OR Bipolar OR Mania) AND (Topiramate OR Topamax) AND ("Randomized Clinical Trial" OR RCT) = **5**

("Bipolar Disorder"[Mesh] OR Bipolar OR Mania) AND (Lamotrigine OR Lamictal) AND ("Randomized Clinical Trial" OR RCT) = **12**

("Bipolar Disorder"[Mesh] OR Bipolar OR Mania) AND (Metoprolol OR Lopressor OR Toprol) AND ("Randomized Clinical Trial" OR RCT) = 0

("Bipolar Disorder"[Mesh] OR Bipolar OR Mania) AND (Propranolol OR Indera) AND ("Randomized Clinical Trial" OR RCT) = 0

("Bipolar Disorder"[Mesh] OR Bipolar OR Mania) AND (Atenolol OR Tenormin) AND ("Randomized Clinical Trial" OR RCT) = 0

("Bipolar Disorder"[Mesh] OR Bipolar OR Mania) AND (Bisoprolol OR Zebeta OR Concor) AND ("Randomized Clinical Trial" OR RCT) = 0

("Bipolar Disorder"[Mesh] OR Bipolar OR Mania) AND (Timolol OR Blocadren OR Betimol OR Timoptic) AND ("Randomized Clinical Trial" OR RCT) = 0

("Bipolar Disorder"[Mesh] OR Bipolar OR Mania) AND (Nadolol OR Corgard) AND ("Randomized Clinical Trial" OR RCT) = 0

("Bipolar Disorder"[Mesh] OR Bipolar OR Mania) AND (Candesartan OR Atacand) AND ("Randomized Clinical Trial" OR RCT) = 0

("Bipolar Disorder"[Mesh] OR Bipolar OR Mania) AND (Lisinopril OR Zestril OR Prinivil) AND ("Randomized Clinical Trial" OR RCT) = 0

("Bipolar Disorder"[Mesh] OR Bipolar OR Mania) AND (Flunarizine OR Sibelium) AND ("Randomized Clinical Trial" OR RCT) = 0

("Bipolar Disorder"[Mesh] OR Bipolar OR Mania) AND (Amitriptyline OR Elavil OR Endep OR Tryptanol) AND ("Randomized Clinical Trial" OR RCT) = 0

("Bipolar Disorder"[Mesh] OR Bipolar OR Mania) AND (Venlafaxine OR Effexor OR Efexor) AND ("Randomized Clinical Trial" OR RCT) = **7**

("Bipolar Disorder"[Mesh] OR Bipolar OR Mania) AND (Erenumab OR Aimovig) AND ("Randomized Clinical Trial" OR RCT) = 0

("Bipolar Disorder"[Mesh] OR Bipolar OR Mania) AND (Fremanezumab OR Ajovy) AND ("Randomized Clinical Trial" OR RCT) = 0

("Bipolar Disorder"[Mesh] OR Bipolar OR Mania) AND (Galcanezumab OR Emgality) AND ("Randomized Clinical Trial" OR RCT) = 0

("Bipolar Disorder"[Mesh] OR Bipolar OR Mania) AND (Eptinezumab OR Vyepti) AND ("Randomized Clinical Trial" OR RCT) = 0

("Bipolar Disorder"[Mesh] OR Bipolar OR Mania) AND (OnabotulinumtoxinA OR Botox) AND ("Randomized Clinical Trial" OR RCT) = 0

("Bipolar Disorder"[Mesh] OR Bipolar OR Mania) AND (Lidocaine OR Xylocaine OR Lignocaine) AND ("Randomized Clinical Trial" OR RCT) = 0

("Bipolar Disorder"[Mesh] OR Bipolar OR Mania) AND (Bupivacaine OR Marcaine OR Sensorcaine OR Exparel) AND ("Randomized Clinical Trial" OR RCT) = 0

("Bipolar Disorder"[Mesh] OR Bipolar OR Mania) AND (Methylprednisolone OR Medrol) AND ("Randomized Clinical Trial" OR RCT) = 0

("Bipolar Disorder"[Mesh] OR Bipolar OR Mania) AND (Dexamethasone OR Decadron OR Ozurdex OR Maxidex) AND ("Randomized Clinical Trial" OR RCT) = 0

("Bipolar Disorder"[Mesh] OR Bipolar OR Mania) AND (Atogepant OR Qulipta) AND ("Randomized Clinical Trial" OR RCT) = 0

("Bipolar Disorder"[Mesh] OR Bipolar OR Mania) AND (Rimegepant OR Nurtec) AND ("Randomized Clinical Trial" OR RCT) = 0

Strategy #3

"Migraine Disorders"[Mesh] OR Migraine

("Migraine Disorders"[Mesh] OR Migraine) AND (Valproic acid OR Valproate OR Divalproex) AND ("Randomized Clinical Trial" OR RCT) = **24**

("Migraine Disorders"[Mesh] OR Migraine) AND (Lithium) AND ("Randomized Clinical Trial" OR RCT) = 0

("Migraine Disorders"[Mesh] OR Migraine) AND (Quetiapine OR Seroquel) AND ("Randomized Clinical Trial" OR RCT) = 0

("Migraine Disorders"[Mesh] OR Migraine) AND (Asenapine OR Saphris OR Sycrest) AND ("Randomized Clinical Trial" OR RCT) = 0

("Migraine Disorders"[Mesh] OR Migraine) AND (Aripiprazole OR Abilify OR Aristada) AND ("Randomized Clinical Trial" OR RCT) = 0

("Migraine Disorders"[Mesh] OR Migraine) AND (Paliperidone OR Invega) AND ("Randomized Clinical Trial" OR RCT) = 0

("Migraine Disorders"[Mesh] OR Migraine) AND (Risperidone OR Risperdal) AND ("Randomized Clinical Trial" OR RCT) = 0

("Migraine Disorders"[Mesh] OR Migraine) AND (Cariprazine OR Vraylar) AND ("Randomized Clinical Trial" OR RCT) = 0

("Migraine Disorders"[Mesh] OR Migraine) AND (Olanzapine OR Zyprexa OR Zydis) AND ("Randomized Clinical Trial" OR RCT) = 0

("Migraine Disorders"[Mesh] OR Migraine) AND (Carbamazepine OR Tegretol OR Equetro OR Carbatrol OR Epitol) AND ("Randomized Clinical Trial" OR RCT) = **6**

("Migraine Disorders"[Mesh] OR Migraine) AND (Ziprasidone OR Geodon) AND ("Randomized Clinical Trial" OR RCT) = 0

("Migraine Disorders"[Mesh] OR Migraine) AND (Haloperidol OR Haldol) AND ("Randomized Clinical Trial" OR RCT) = **2**

("Migraine Disorders"[Mesh] OR Migraine) AND (Chlorpromazine OR Thorazine OR Largactil) AND ("Randomized Clinical Trial" OR RCT) = **2**

("Migraine Disorders"[Mesh] OR Migraine) AND (Clozapine OR Clozaril OR Leponex OR Fazaclo OR Versacloz) AND ("Randomized Clinical Trial" OR RCT) = 0

("Migraine Disorders"[Mesh] OR Migraine) AND (Tamoxifen OR Nolvadex OR Soltamox) AND ("Randomized Clinical Trial" OR RCT) = **1**

COCHRANE SEARCHES

Strategy #1

("Bipolar Disorder"[Mesh] OR Bipolar OR Mania) AND ("Migraine Disorders"[Mesh] OR Migraine) AND ("Randomized Clinical Trial" OR RCT) = 1

Strategy #2

"Bipolar Disorder"[Mesh] OR Bipolar OR Mania

("Bipolar Disorder"[Mesh] OR Bipolar OR Mania) AND (Valproic acid OR Valproate OR Divalproex) AND ("Randomized Clinical Trial" OR RCT) = **40**

("Bipolar Disorder"[Mesh] OR Bipolar OR Mania) AND (Topiramate OR Topamax) AND ("Randomized Clinical Trial" OR RCT) = **5**

("Bipolar Disorder"[Mesh] OR Bipolar OR Mania) AND (Lamotrigine OR Lamictal) AND ("Randomized Clinical Trial" OR RCT) = **14**

("Bipolar Disorder"[Mesh] OR Bipolar OR Mania) AND (Metoprolol OR Lopressor OR Toprol) AND ("Randomized Clinical Trial" OR RCT) = 0

("Bipolar Disorder"[Mesh] OR Bipolar OR Mania) AND (Propranolol OR Indera) AND ("Randomized Clinical Trial" OR RCT) = 0

("Bipolar Disorder"[Mesh] OR Bipolar OR Mania) AND (Atenolol OR Tenormin) AND ("Randomized Clinical Trial" OR RCT) = 0

("Bipolar Disorder"[Mesh] OR Bipolar OR Mania) AND (Bisoprolol OR Zebeta OR Concor) AND ("Randomized Clinical Trial" OR RCT) = 0

("Bipolar Disorder"[Mesh] OR Bipolar OR Mania) AND (Timolol OR Blocadren OR Betimol OR Timoptic) AND ("Randomized Clinical Trial" OR RCT) = 0

("Bipolar Disorder"[Mesh] OR Bipolar OR Mania) AND (Nadolol OR Corgard) AND ("Randomized Clinical Trial" OR RCT) = 0

("Bipolar Disorder"[Mesh] OR Bipolar OR Mania) AND (Candesartan OR Atacand) AND ("Randomized Clinical Trial" OR RCT) = 0

("Bipolar Disorder"[Mesh] OR Bipolar OR Mania) AND (Lisinopril OR Zestril OR Prinivil) AND ("Randomized Clinical Trial" OR RCT) = 0

("Bipolar Disorder"[Mesh] OR Bipolar OR Mania) AND (Flunarizine OR Sibelium) AND ("Randomized Clinical Trial" OR RCT) = 0

("Bipolar Disorder"[Mesh] OR Bipolar OR Mania) AND (Amitriptyline OR Elavil OR Endep OR Tryptanol) AND ("Randomized Clinical Trial" OR RCT) = 0

("Bipolar Disorder"[Mesh] OR Bipolar OR Mania) AND (Venlafaxine OR Effexor OR Efexor) AND ("Randomized Clinical Trial" OR RCT) = **6**

("Bipolar Disorder"[Mesh] OR Bipolar OR Mania) AND (Erenumab OR Aimovig) AND ("Randomized Clinical Trial" OR RCT) = 0

("Bipolar Disorder"[Mesh] OR Bipolar OR Mania) AND (Fremanezumab OR Ajovy) AND ("Randomized Clinical Trial" OR RCT) = 0

("Bipolar Disorder"[Mesh] OR Bipolar OR Mania) AND (Galcanezumab OR Emgality) AND ("Randomized Clinical Trial" OR RCT) = 0

("Bipolar Disorder"[Mesh] OR Bipolar OR Mania) AND (Eptinezumab OR Vyepti) AND ("Randomized Clinical Trial" OR RCT) = 0

("Bipolar Disorder"[Mesh] OR Bipolar OR Mania) AND (OnabotulinumtoxinA OR Botox) AND ("Randomized Clinical Trial" OR RCT) = 0

("Bipolar Disorder"[Mesh] OR Bipolar OR Mania) AND (Lidocaine OR Xylocaine OR Lignocaine) AND ("Randomized Clinical Trial" OR RCT) = 0

("Bipolar Disorder"[Mesh] OR Bipolar OR Mania) AND (Bupivacaine OR Marcaine OR Sensorcaine OR Exparel) AND ("Randomized Clinical Trial" OR RCT) = 0

("Bipolar Disorder"[Mesh] OR Bipolar OR Mania) AND (Methylprednisolone OR Medrol) AND ("Randomized Clinical Trial" OR RCT) = 0

("Bipolar Disorder"[Mesh] OR Bipolar OR Mania) AND (Dexamethasone OR Decadron OR Ozurdex OR Maxidex) AND ("Randomized Clinical Trial" OR RCT) = 0

("Bipolar Disorder"[Mesh] OR Bipolar OR Mania) AND (Atogepant OR Qulipta) AND ("Randomized Clinical Trial" OR RCT) = 0

("Bipolar Disorder"[Mesh] OR Bipolar OR Mania) AND (Rimegepant OR Nurtec) AND ("Randomized Clinical Trial" OR RCT) = 0

Strategy #3

"Migraine Disorders"[Mesh] OR Migraine

("Migraine Disorders"[Mesh] OR Migraine) AND (Valproic acid OR Valproate OR Divalproex) AND ("Randomized Clinical Trial" OR RCT) = **20**

("Migraine Disorders"[Mesh] OR Migraine) AND (Lithium) AND ("Randomized Clinical Trial" OR RCT) = 0

("Migraine Disorders"[Mesh] OR Migraine) AND (Quetiapine OR Seroquel) AND ("Randomized Clinical Trial" OR RCT) = 0

("Migraine Disorders"[Mesh] OR Migraine) AND (Asenapine OR Saphris OR Sycrest) AND ("Randomized Clinical Trial" OR RCT) = 0

("Migraine Disorders"[Mesh] OR Migraine) AND (Aripiprazole OR Abilify OR Aristada) AND ("Randomized Clinical Trial" OR RCT) = 0

("Migraine Disorders"[Mesh] OR Migraine) AND (Paliperidone OR Invega) AND ("Randomized Clinical Trial" OR RCT) = 0

("Migraine Disorders"[Mesh] OR Migraine) AND (Risperidone OR Risperdal) AND ("Randomized Clinical Trial" OR RCT) = 0

("Migraine Disorders"[Mesh] OR Migraine) AND (Cariprazine OR Vraylar) AND ("Randomized Clinical Trial" OR RCT) = 0

("Migraine Disorders"[Mesh] OR Migraine) AND (Olanzapine OR Zyprexa OR Zydis) AND ("Randomized Clinical Trial" OR RCT) = 0

("Migraine Disorders"[Mesh] OR Migraine) AND (Carbamazepine OR Tegretol OR Equetro OR Carbatrol OR Epitol) AND ("Randomized Clinical Trial" OR RCT) = **1**

("Migraine Disorders"[Mesh] OR Migraine) AND (Ziprasidone OR Geodon) AND ("Randomized Clinical Trial" OR RCT) = 0

("Migraine Disorders"[Mesh] OR Migraine) AND (Haloperidol OR Haldol) AND ("Randomized Clinical Trial" OR RCT) = **2**

("Migraine Disorders"[Mesh] OR Migraine) AND (Chlorpromazine OR Thorazine OR Largactil) AND ("Randomized Clinical Trial" OR RCT) = **3**

("Migraine Disorders"[Mesh] OR Migraine) AND (Clozapine OR Clozaril OR Leponex OR Fazaclo OR Versacloz) AND ("Randomized Clinical Trial" OR RCT) = 1

("Migraine Disorders"[Mesh] OR Migraine) AND (Tamoxifen OR Nolvadex OR Soltamox) AND ("Randomized Clinical Trial" OR RCT) = 0

SUPPLEMENTAL MATERIAL – DETAILED SEARCHES RESULTS

Total results in the search for **comorbid bipolar disorder and migraine** = 1

EXCLUDED = 1 (REVIEW)

Total results in the search for **bipolar disorder** = 126

Results in the search for migraine drugs in the treatment of bipolar disorder after removing duplicates = 65

INCLUDED = 8

EXCLUDED = 57

EXCLUDED – REVIEW = 25

EXCLUDED – NOT BIPOLAR DISORDER = 4

EXCLUDED – PHARMACOECONOMIC = 2

EXCLUDED – NO SINGLE-ARM DRUG EFFICACY = 21

EXCLUDED – NO EFFICACY ASSESSMENT ON BD SYMPTOMS = 4

EXCLUDED – CASE REPORT = 1

Included by reference analysis = 7

Total included = 15

Total results in the search for **Migraine** = 61

Results in the search for bipolar disorder drugs in the treatment of migraine after removing duplicates = 35

INCLUDED = 16

EXCLUDED = 19

EXCLUDED – REVIEW = 14

EXCLUDED – NO EFFICACY ASSESSMENT ON MIGRAINE SYMPTOMS = 3

EXCLUDED – NO SINGLE-ARM DRUG EFFICACY = 2

Included by reference analysis = 1

Total included = 17
